# Supplementary material for: Favour the best in case of emergency cricothyroidotomy–a randomized cross-over trial on manikin focused training and simulation of common devices
Source: PeerJ. 2024 Aug 23;12:e17788. doi: 10.7717/peerj.17788 (PMC11348895; doi:10.7717/peerj.17788)
Supplement: Supplemental Information 2 [file peerj-12-17788-s002.docx]

**Simulation study on the Cricothyroidotomy Training Phantom**

'Evaluation and Comparison of Three Different Cricothyroidotomy Techniques (ScalpelCric Study)'

| Title | Evaluation of the new ScalpelCric set for surgical cricothyroidotomy ('tracheostomy') according to the Scalpel-Bougie technique and comparison with two alternative cricothyroidotomy techniques on the training phantom (A) and comparison of the new ScalpelCric set with conventional surgical tracheotomy on a pig trachea (B) Influence of training (A+B) in a simulation scenario (C) |
| --- | --- |
| Short Title | ScalpelCric-Study |
| Principal investigator | Dr. med. Nicole Didion |
| Indication | A) Evaluation and Comparison of Three Different Cricothyroidotomy Techniques on the Training Phantom: 1.) Scalpel-Bougie Technique with the ScalpelCric Set 2.) Surgical Technique with Scalpel and Speculum 3.) Seldinger Technique with the Melker Emergency Cricothyroidotomy Catheter Set B) Evaluation and Comparison of Two Different Cricothyroidotomy Techniques on a Pig Trachea: 1.) Scalpel-Bougie Technique with the ScalpelCric Set 2.) Surgical Technique with Scalpel and Speculum C) Influence of Training (A+B) on the Time for Cricothyroidotomy Indication and Its Impact on the Technique Selection (n=26) |
| Primary objective and endpoint | Time for successful cricothyroidotomy |
| Secondary objectives and endpoints | 1. Success rate on the first attempt. 2. Number of attempts for a successful cricothyroidotomy. 3. Extent of tracheal trauma (in mm). 4. User assessment (rated on a Likert scale from 1 to 4). 5. Impact of training on the time for cricothyroidotomy indication. 6. Impact of training on the selection of technique |
| Study Design | Monocentric, prospective, randomized data collection. |
| Study Population | A) Staff from the Department of Anaesthesiology at the University Medical Center Mainz:   - 50 board-certified Anaesthesiologists with additional certification in Emergency Medicine - 50 Anaesthesiology Residents - 50 Paramedics, Emergency Medical Technicians and Emergency Medical Services Practitioner - 50 Medical Students   B) Staff from the Department of Anaesthesiology at the University Medical Center Mainz:   - 10 board-certified Anaesthesiologists regularly deployed on rescue helicopters, emergency medical service vehicles, and intra-hospital emergency teams - 10 Anaesthesiology Residents deployed on emergency medical service vehicles and intra-hospital emergency teams   C) Staff from the Department of Anaesthesiology at the University Medical Center Mainz (Residents and Board-certified Anaesthesiologists) participating in the Airway Workshop organized by the Department of Anaesthesiology at the University Medical Center Mainz. |
| Study duration | Data collection: November 2018 - January 2019 |
| Number of participants | 1. 200 2. 20 3. 26 |
| Statistical procedure | 1. Hypothesis:    1. Superiority of the ScalpelCric technique and the surgical technique over the Seldinger technique with the Melker Set    2. Equivalence of the ScalpelCric technique and the surgical technique with a tolerance range of +/- 7 seconds median time.   Primary Endpoint:   - Hypothesis Test: Survival analysis with Cox regression using robust sandwich estimator for variance clustering. Hypothesis a) Pairwise group comparisons of estimated marginal means using Wald tests with alpha adjustment according to Tukey. Hypothesis b) Pairwise group comparison with equivalence test using two one-sided 95% confidence intervals for the difference and checking if the combined interval contains the tolerance interval. - Descriptive: Kaplan-Meier curves separated by cricothyroidotomy technique.   Secondary Endpoint 1:   - Hypothesis Test: Generalized linear mixed model (binary logistic regression). - Descriptive: Absolute and relative frequencies separated by cricothyroidotomy technique overall and stratified by employee group.   Secondary Endpoint 2:   - Hypothesis Test: Generalized linear mixed model (ordinal logistic regression). - Descriptive: Absolute and relative frequencies separated by cricothyroidotomy technique overall and stratified by employee group.   Secondary Endpoint 3:   - Hypothesis Test: Linear mixed model (linear regression). - Descriptive: Mean, median, range, and standard deviation separated by cricothyroidotomy technique overall and stratified by employee group.   Secondary Endpoint 4:   - Hypothesis Test: Generalized linear mixed model (ordinal logistic regression). - Descriptive: Absolute and relative frequencies separated by cricothyroidotomy technique overall and stratified by employee group.   Variables in all regression models: Employee group, cricothyroidotomy technique.  B)  Hypothesis: Superiority of the ScalpelCric technique over the Seldinger technique with the Melker Set.  Primary Endpoint:   - Hypothesis Test: Same as A) Primary Endpoint. - Descriptive: Same as A) Primary Endpoint.   Secondary Endpoint 1:   - Hypothesis Test: McNemar's Test. - Descriptive: Same as A) Secondary Endpoint 1.   Secondary Endpoint 2:   - Hypothesis Test: Page Trend Test. - Descriptive: Same as A) Secondary Endpoint 2.   Secondary Endpoint 3:   - Hypothesis Test: t-Test for paired samples. - Descriptive: Same as A) Secondary Endpoint 3.   Secondary Endpoint 4:   - Hypothesis Test: Page Trend Test. - Descriptive: Same as A) Secondary Endpoint 4.   C)  Secondary Endpoint 5:   - Hypothesis Test: Log-rank Test. - Descriptive: Kaplan-Meier representation.   Secondary Endpoint 6:   - Hypothesis Test: Chi-square test for equality of distributions in two independent groups. - Descriptive: Absolute and conditional relative frequencies.   Significance threshold in all tests: p < 0.05.  Sample size: Based on a study by Ott et al. (2018, p.23), the effect of surgical technique vs. Seldinger technique is estimated for a median time to success of 52s vs. 73s (with exponentially distributed survival times HR 1.4). The same effect is assumed for the ScalpelCric technique. For further assumptions and details on sample size calculation, see below.  A)  a) With a sample size of 25 doctors in each of the 5 experience groups, the power for the pairwise Wald tests for superiority is approximately 95% at the alpha level of 0.05 with alpha adjustment according to Tukey. With 25 doctors per group, a smaller effect of a median time of 58s vs. 73s (HR 1.25 with exponentially distributed times) can be detected with a power of approximately 80%. A power of approximately 80% is already achieved with 16 doctors per experience group if the effect is a time of 52s vs. 73s median.  b) The equivalence test with a tolerance range of +/- 7s has a power of approximately 30% with 25 doctors per group, approximately 70% with 43 doctors per group, and approximately 80% with 50 doctors per group.  B) With a sample size of 20 doctors in both experience groups, the power for the test of superiority is approximately 77%, analogous to A).  Statistical analysis is conducted using R (Version 3.5.1, R Core Team, Vienna, 2018) |

Materials:

• ScalpelCric Set (Company: VBM Medizintechnik GmbH, Sulz, Germany)

• Scalpel and Speculum: Scalpel - Feather® No 11 (Company: Feather® Safety Razor Co. Ltd, Osaka, Japan) Speculum - Medicon Instrumente 14cm (Medicon eG, Tuttlingen, Germany) • Endotracheal Tube - Rüschelit® Super Safety Clear ID 5.0mm (Teleflex® Medical GmbH, Fellbach, Germany)

• Melker Emergency Cricothyroidotomy Catheter Set (Company: Cook® Medical Inc., Bloomington, USA)

• Crico-Trainer "Adelaide" including two-layered skin and trachea (Company: VBM Medizintechnik GmbH, Sulz, Germany)

• Crico-Trainer "Pig Trachea" (Company: VBM Medizintechnik GmbH, Sulz, Germany)

• Pig Trachea

• Stopwatch

• Case Report Forms (CRF Forms)

**Study course:**

• Theoretical introduction to the techniques (instructional video 5 minutes)

• Practical training on the training phantom (1 attempt per technique)

• Documentation of demographic participant data

• Randomization using an established computer-based program (QuickCalcs, GraphPad Software) by the study center

- Definition of Endpoints:
- Primary Endpoint:
- Time for successful cricothyroidotomy: from touching the equipment to positioning the endotracheal tube in the trachea. Endoscopic confirmation of placement is done using the aScope4 (Ambu GmbH, Bad Nauheim, Germany).
- Secondary Endpoints:

•Success rate on the first attempt

• Number of attempts

• Extent of tracheal trauma (horizontal and vertical in mm)

• User assessment (Likert Scale: 1 = very easy, 2 = easy, 3 = difficult, 4 = very difficult)

• Impact of training on the time for cricothyroidotomy indication

• Impact of training on the selection of technique

**Emergency Cricothyroidotomy: (Recommendation for Prehospital Airway Management from the Scientific Working Group on Emergency Medicine of the German Society of Anaesthesiology and Intensive Care Medicine and the Airway Management Commission of the German Society of Anaesthesiology).**

Airway management is a core competency of anesthesiologists, intensive care physicians, and emergency physicians. Without secured or open airways, effective oxygenation and ventilation are not possible. Prehospital airway management is significantly more challenging compared to in-hospital conditions due to numerous factors such as patient condition, environmental conditions, limited equipment, and individual knowledge of medical personnel.

Surgical airway securing is at the end of the airway management algorithm as the "last resort" but can also be employed primarily, for example, in cases of upper airway or glottic obstruction. In this procedure, the cricothyroid ligament is incised, and a cannula or tube is inserted below the level of the glottis.

There are three fundamental techniques:

1. **"Catheter-over-needle technique":** In this method, airway cannulation is performed similarly to placing a peripheral intravenous catheter.
2. **"Seldinger technique":** The airway is punctured through the cricothyroid ligament with a needle, a guide wire is introduced through it, and the tracheal tube is then inserted over the wire.
3. **"Surgical Cricothyroidotomy":** In this technique, after a skin incision, the cricothyroid ligament is incised with a scalpel. The thyroid and cricoid cartilages are separated, for example, using a speculum, and a tracheal tube or a thin endotracheal tube can be introduced into the trachea.

A variant of the surgical technique is the Scalpel-Bougie technique. Similar to surgical cricothyroidotomy, a skin incision is made with a scalpel. Then, the scalpel is rotated, and the bougie is introduced into the created access. The tube can be advanced over the inserted bougie. In case of tube insertion failure, oxygenation can alternatively be performed through the bougie. The ScalpelCric Set (VBM, Sulz) was designed for this technique


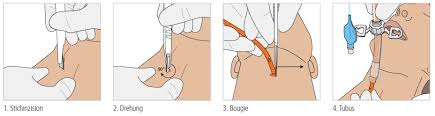


**Numerous studies on patients, human cadavers, and animal models yield highly contradictory results regarding the successful execution of cricothyroidotomy. Both the personal experience of subjects in airway management and the overall medical education level (physician, nurse, medical student, or paramedic) are highly heterogeneous, and the case numbers are often very low. As a result, an evidence-based recommendation for the optimal cricothyroidotomy technique cannot be derived at the present time. In this present study, the ScalpelCric will be examined for the first time**

**CE-Zertifikate:**

**
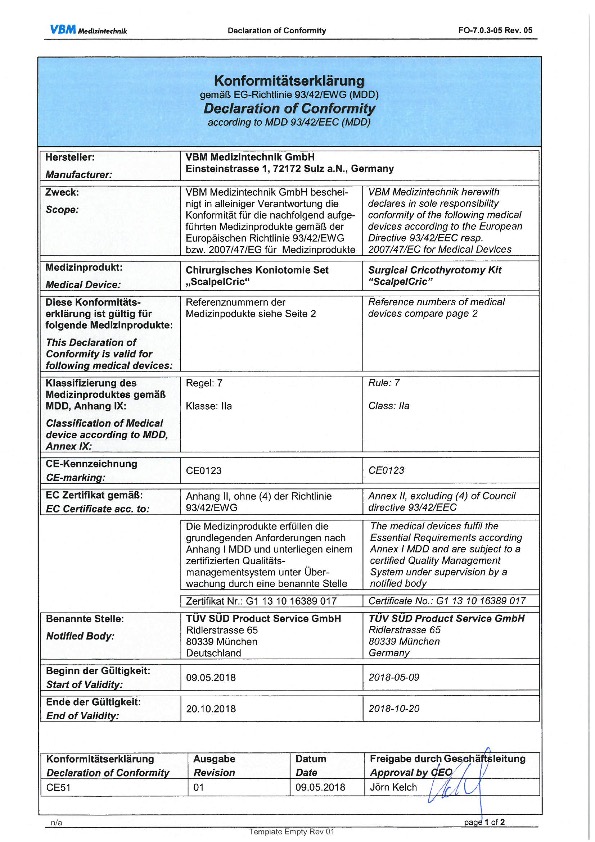
**
